# The Combination of Random Mutagenesis and Sequencing Highlight the Role of Unexpected Genes in an Intractable Organism

**DOI:** 10.1371/journal.pgen.1004895

**Published:** 2015-01-08

**Authors:** Damien Faivre, Jens Baumgartner

**Affiliations:** Max Planck Institute of Colloids and Interfaces, Science Park Golm, Potsdam, Germany; Indiana University, United States of America

A large variety of organisms are capable of synthesizing hard matter in a process called biomineralization [1]. The transformation of a genetic blueprint into minerals such as, for example, calcium phosphate in bones and calcium carbonate in eggs or seashells provides a mechanical support for organismic growth and protection against predators, respectively. Iron oxides formed by fishes and birds provide them with magnetic properties used for magnetoreception and orientation [2], [3]. The biomineralization processes are remarkable for numerous reasons: organisms, contrary to engineers, have to form these biological materials with a limited subset of biologically available chemical elements and at physiological conditions. Still, these reduced means are not at the detriment of their function, which often surpasses man-made materials based on equivalent elements [4]. Therefore, understanding how biomineralizing organisms process chemical elements based on their genetic program is of primary interest. However, the biological mechanisms behind biomineralization have remained unclear, partly because of limited genetic knowledge: model organisms are limited to a few unicellular organisms [5], [6]. Therefore, the question has arisen of what genetic approach to use to get genetic information about the large majority of organisms that have remained intractable.

## Magnetotactic Bacteria: Simply Microorganisms, but Not So Simple

The recent advances in sequencing techniques now offer the opportunity to bypass some of the restrictions associated with the unavailability of genetic systems to get novel insights into important microbial processes such as those associated with biomineralization. In their study, Rahn-Lee et al. [7] combine established genetic techniques (random mutagenesis) with modern sequencing platforms to understand magnetite biomineralization in the magnetotactic bacteria *Desulfovibrio magneticus* RS-1. Magnetotactic bacteria are microorganisms able to form intracellular magnetic nanoparticles made of the iron oxide magnetite (Fe_3_O_4_) or the iron sulfide greigite (Fe_3_S_4_) [8]. The nanoparticles together with their membrane envelope are called magnetosomes. They have strain-specific sizes and morphologies and are typically arranged in chains in order to form a magnetic dipole strong enough to passively orient the bacteria along the magnetic field lines of the Earth, a process called magnetotaxis [9].

**Figure 1:**
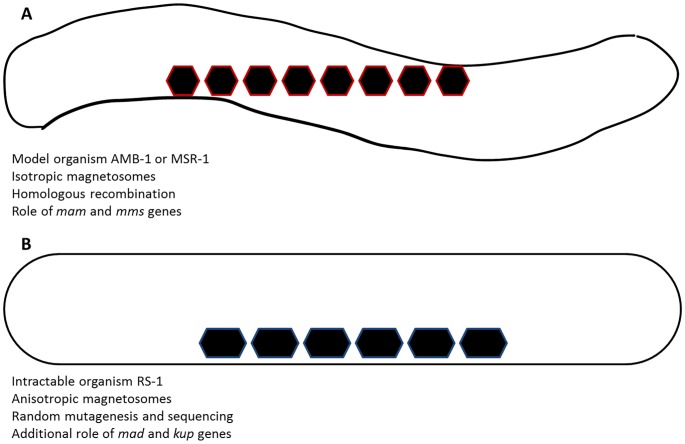
Sketch of magnetite biomineralization and associated differences. In the model organisms (magnetospirilla, AMB-1 or MSR-1), isotropic magnetosomes are produced. Genetic studies have highlighted the roles of *mam* and *mms* genes in the process (A). In turn, intractable organisms such as RS-1, where elongated magnetosomes are produced, could so far not be genetically studied. By random mutagenesis and whole-genome sequencing, Rahn-Lee et al. (2015) showed the additional role of *kup* and *mad* genes in the process, possibly in their morphology control of the nanoparticles (B).

The genomes of numerous strains have been sequenced [10]. However, genetic tools permitting the manipulation of the microorganisms are only available for two magnetospirilla strains: *Magnetospirillum gryphiswaldense* [11] and *M. magneticum* [12]. In these strains, the magnetosomes are formed and arranged thanks to a subset of genes called the magnetosome island [13], which, in particular, encompasses the *mamAB*, *mamGFDC*, *mms6*, and *mamXY* operons (Fig. 1A) [14], [15]. However, RS-1, the strain studied by Rahn-Lee et al. [7], forms elongated magnetosomes in contrast to magnetospirilla, which form cubooctaedral magnetosomes. Therefore, RS-1 would be a model organism to use to study the functional diversity of compartmentalization and biomineral formation in magnetotactic bacteria, providing tools were available to do so.

## Getting Genetic Information in an Intractable Organism

The authors used random mutagenesis to generate nonmagnetic mutants and combined it with whole-genome re-sequencing to identify the mutated genes. In particular, Rahn-Lee et al. [7] first cultivated RS-1 in conditions where the microorganism no longer formed magnetosomes and then performed UV and chemical mutagenesis. Since screening a large number of colonies was impractical, they employed a two-step strategy that consisted of first selecting in liquid to increase the proportion of non-magnetic cells in the population and then only screening for single colonies of non-magnetic phenotypes [7]. These colonies of no- or low-magnetism were then analyzed by whole-genome sequencing to determine the causative genetic change. After the mutation for each strain was identified, the authors used PCR and Sanger sequencing to check for this change in the other strains isolated from the same outgrowth and analyzed those strains that were not clones by whole-genome sequencing to determine their mutation.

This approach led to the isolation of about 30 mutants, with mutations in genes shared amongst all magnetotactic bacteria, but also, more interestingly, with *mad* genes that are unique to the magnetotactic δ-proteo bacteria and even genes potentially unique to RS-1. The group of A. Komeili found that a potassium transporter (*kup*) is important for biomineralization of magnetite (Fig. 1B) [7], a surprising discovery since there is a priori no reason to expect the involvement of potassium in an iron oxide mineral. The authors, in addition, presented the first experimental proof of the involvement of *mad* genes in the control of the magnetosome morphology. This is an important confirmation since a bioinformatic study proposed earlier that these so-called *mad* genes could be responsible for the morphology control observed in some strains, since these genes are specifically found in magnetotactic δ-proteobacteria-forming elongated magnetosomes such as RS-1, and not in magnetospirilla [10].

In conclusion, the general methodology presented here will be of immediate relevance to other scientists working with fastidious and genetically intractable organisms, not limited to biomineralizing ones. In addition, the study delivers significant advancements for the understanding of biomineralization and its variety in prokaryotes by presenting the first genetic analysis of magnetotactic bacteria outside of the commonly studied α-proteobacteria. However, as random mutagenesis is stochastic and not directed, important genes might remain unprobed and, therefore, their role might possibly be overlooked by this method. Therefore, efforts in the development of genetic tools should not be abandoned. In addition, complementation of this approach by physical and chemical analytical techniques in the near future will enable the complete multidisciplinary understanding of biomineralization in different strains of magnetotactic bacteria.
